# Comparison of bioavailability between the most available generic tablet formulation containing artemether and lumefantrine on the Tanzanian market and the innovator’s product

**DOI:** 10.1186/1475-2875-12-174

**Published:** 2013-05-30

**Authors:** Omary MS Minzi, Ignace A Marealle, Seif Shekalaghe, Omar Juma, Eliford Ngaimisi, Mwajuma Chemba, Mastidia Rutaihwa, Salim Abdulla, Philip Sasi

**Affiliations:** 1 Unit of Pharmacology and Therapeutics, School of Pharmacy, Muhimbili University of Health and Allied Sciences, PO BOX 65013, Dar Es Salaam, Tanzania; 2 Ifakara Health Institute, PO Box 78373, Dar es Salaam, Tanzania; 3 Department of Clinical Pharmacology, School of Medicine, Muhimbili University of Health and Allied Sciences, PO BOX 65010, Dar Es Salaam, Tanzania

**Keywords:** Bioavailability, Bioequivalence, Artefan, Coartem, Generics

## Abstract

**Background:**

Existence of anti-malarial generic drugs with low bioavailability marketed on sub-Saharan Africa raises a concern on patients achieving therapeutic concentrations after intake of such products. This work compared bioavailability of one generic tablet formulation with innovator’s product. Both were fixed dose combination tablet formulations containing artemether and lumefantrine.

**Methodology:**

The study was conducted in Dar Es Salaam, Tanzania, in which a survey of the most abundant generic containing artemether-lumefantrine tablet formulation was carried out in retail pharmacies. The most widely available generic (Artefan®, Ajanta Pharma Ltd, Maharashtra, India) was sampled for bioavailability comparison with Coartem® (Novartis Pharma, Basel, Switzerland) - the innovator’s product. A randomized, two-treatment cross-over study was conducted in 18 healthy Tanzanian black male volunteers. Each volunteer received Artefan® (test) and Coartem® (as reference) formulation separated by 42 days of drug-free washout period. Serial blood samples were collected up to 168 hours after oral administration of a single dose of each treatment. Quantitation of lumefantrine plasma levels was done using HPLC with UV detection. Bioequivalence of the two products was assessed in accordance with the US Food and Drug Authority (FDA) guidelines.

**Results:**

The most widely available generic in pharmacies was Artefan® from India. All eighteen enrolled volunteers completed the study and both test and reference tablet formulations were well tolerated. It was possible to quantify lumefantrine alone, therefore, the pharmacokinetic parameters reported herein are for lumefantrine. The geometric mean ratios for C_max_, AUC_0-t_ and AUC_0-∞_ were 84% in all cases and within FDA recommended bioequivalence limits of 80% – 125%, but the 90% confidence intervals were outside FDA recommended limits (CI 49–143%, 53 - 137%, 52 - 135% respectively). There were no statistical significant differences between the two formulations with regard to PK parameters (P > 0.05).

**Conclusions:**

Although the ratios of AUCs and C_max_ were within the acceptable FDA range, bioequivalence between Artefan® and Coartem® tablet formulations was not demonstrated due to failure to comply with the FDA 90% confidence interval criteria. Based on the observed total drug exposure (AUCs), Artefan® is likely to produce a similar therapeutic response as Coartem®.

## Background

Malaria is the major cause of morbidity and mortality in the sub-Saharan Africa (SSA) especially in children below five years of age. Chemotherapy is the cornerstone in the management of malaria in endemic areas. Due to high resistance rates against sulphadoxine-pyrimethamine (SP), artemisinin-based combination therapy (ACT) containing artemether-lumefantrine (Alu) replaced SP as first-line drug of choice for treatment of uncomplicated falciparum malaria in many countries of SSA, including Tanzania.

The change of malaria treatment guidelines has been accompanied by infiltration of generic Alu tablet formulation in the health systems of these countries. High prices of branded products e.g. Coartem® calls for alternative cheaper generic products. Generic formulations are promoted to address accessibility and affordability. Unfortunately, there have been disappointing reports of marketing anti-malarial generic products that are fake, substandard and with low bioavailability in South East Asia [1], SSA and East Africa including Kenya and Tanzania [2-8]. The use of poor quality anti-malarial drugs threatens the life span of ACT and may result in sub-therapeutic concentrations leading to poor treatment outcomes and emergence of drug resistance. Reports on impaired clinical response of uncomplicated falciparum malaria due to use of poor-quality artemether and decreased sensitivity of the parasite on artesunate were recently published [9-11]. Already, there are sporadic reports of resistance against ACT in some malarious regions, creating a concern on the longevity of the currently available and effective anti-malarial drugs in the SSA [12-14].

This paper reports a comparative bioavailability findings between the most available generic tablet formulation containing artemether and lumefantrine (Artefan®) *(*Ajanta Pharma Ltd-India) and Coartem® (Novartis, Basel, Switzerland) as innovator’s product.

## Methodology

### Determination of the most prevalent generic

The study was conducted between February and April 2012. It was preceded by a cross-sectional survey to reveal the most abundant and prescribed generic formulation containing a fixed combination of Alu tablet on the Dar es Salaam market. Among others, Artefan® was found to be abundant and thereafter was subjected to bioequivalence study using Coartem® as comparator.

### Sampling of tablets

Two hundred and twenty four tablets of Artefan® (test formulation), a fixed-dose combination tablets consisting of 20 mg of artemether and 120 mg of lumefantrine (B.No./No.LOT.P0511H, TAN 09.085.PO1BAJA, expiration date July 2013) were purchased from one wholesale pharmacy in Dar es Salaam. The reference formulation Coartem® tablets (F2491, expiration date August 2013) was donated by Dodoma Regional Hospital.

### Clinical study design and ethical clearance

This was a randomized, single dose, open label, single center, two period, two sequence crossover bioequivalence (BE) study. The study protocol was reviewed and approved by the Muhimbili University of Health and Allied Sciences (MUHAS) and Ifakara Health Institute (IHI) institutional review boards. The study was conducted according to Good Clinical Practice. To ensure confidentiality, identification numbers (ID codes) were used instead of volunteers’ names.

### Study area

Recruitment and enrollment of the study volunteers took place at Bagamoyo Clinical Trial Unit in Bagamoyo district. BCTU is part of IHI’s Bagamoyo Research and Training Centre (BRTC). The unit is designed to conduct early phase studies where volunteers can be retained for the entire period of study. The facility has dedicated areas for essential study procedures including volunteer screening, blood sample collection, investigational product preparation, ICU for management of serious adverse events and research wards. Determination of lumefantrine concentration in plasma samples was performed in the MUHAS Bioanalytical laboratory in Dar es Salaam, Tanzania.

### Study participants

Eighteen healthy adult male college students from Bagamoyo Art Institute (TaSUBa) in Coastal region (age 18–35 years) with normal laboratory parameters and willing to provide written informed consent were recruited and enrolled into the study based on the pre-defined inclusion and exclusion criteria.

### Inclusion criteria

a) Healthy male > 18 years of age.

b) Availability during entire study period (two months).

c) Willingness to give written informed consent after being informed of the nature of study.

d) Body mass index (BMI) between 18 and 30 kg/m^2^.

e) No history of anti-malarial drug ingestion in the past one month.

f) Normal Laboratory range parameters from all performed laboratory tests at baseline (FBC, ALT, ASAT, ALP, serum creatinine, total bilirubin and albumin).

g) Must be literate, can speak English and understand written English.

### Exclusion criteria

a) Hypersensitivity to artemether and/or lumefantrine or related compounds.

b) History of conditions that may alter absorption, metabolism, or passage of drugs out of the body (sprue, coeliac disease, Crohn's, colitis, liver, kidney, or thyroid conditions).

c) History of mental illness, drug addiction, drug abuse.

d) A haematocrit value of ≤ 37.0%.

e) Receipt of an investigational drug within four weeks prior to study drug dosing.

f) Currently taking any prescription of systemically acting medications, within seven days prior to study dosing or over-the-counter medication within three days of study dosing.

g) Smoking

h) Positive blood slide for malaria

i) Regular use of barbiturates, carbamazepine, rifampicin, phenytoin, phenothiazines, cimetidine, omeprazole, macrolides, imidazoles, fluoroquinolones and others which can induce or inhibit CYP450.

### Participants recruitment

Recruitment was done through sensitization meetings, which were conducted at the college premises. Permission to conduct sensitization meetings was sought from College administration and thereafter advertisement for the meetings was posted on the college notice board. Interested participants were asked to register their names and invited to attend sensitization meeting where detailed explanation and clarification about the study was given followed by screening of potential subjects in which the potentially eligible participants were identified.

### Screening for eligible participants

Screening was performed to all potential subjects at baseline and based on the inclusion and exclusion criteria to get eligible participants who were then enrolled for the entire study. Baseline screening included taking medical history, performing physical, medical examination and blood laboratory tests. Physical and medical examinations included checking vital signs and performing Electrocardiogram (ECG). Laboratory tests included parasitology (blood slide for malaria parasites), haematology (FBC) and biochemistry (urea, creatinine, total bilirubin, ALT, AST, ALP and albumin).

### Enrollment

Eligible subjects were those who fulfilled the inclusion criteria and none of the exclusion criteria. Out of 31 subjects screened, 18 were enrolled into the study and were randomized into treatment sequence. The participants were advised to abstain from eating grape fruits and drinking grape fruit juice during the entire period of the study [15,16].

### Randomization and study procedures

The eighteen volunteers were randomly assigned to either of the treatment sequence: test period 1, reference period 2; or reference period 1, test period 2. At drug administration, the pharmacist opened a sealed envelope with the corresponding treatment number, which contained the drug allocation for the treatment period, and appropriate drug was administered. The investigators were blinded on the drug, which each volunteer took. The randomization code was kept in the BCTU Data Unit and was decoded (broken) after all the PK analysis had been completed.

### Drug administration, blood sampling and subject follow-up

Each enrolled volunteer received a single adult dose (80/480 mg artemether and lumefantrine respectively) of either Coartem® or Artefan® with a glass of water (200 ml) under supervision of the pharmacist followed by standardized fatty meal. After a 42-day washout period, the volunteers took the alternative drug. On the day of enrolment participants were admitted and retained at the BCTU for three days (72 hours).

Subjects were discharged and returned to BCTU at 168 hours for one blood sample collection and allowed back to college. On each period all subjects underwent clinical evaluations to monitor for adverse drug reactions and assess medication tolerability . This was done at 0.5, 2, 4, 6, 8, 10, 12, 24, 48, 72 and 168 hours post dosing.

### Blood sampling and sample processing

A total of 18 eligible volunteers were enrolled to participate in this study. FDA allows a minimum number of 12 volunteers for bioequivalence study [17,18]. The enrolled volunteers arrived at the BCTU for stay after an overnight fasting. Written informed consent was obtained from each volunteer before participation.

Subjects had a heparinized saline lock placed in an arm to obtain serial venous blood samples for plasma drug concentrations for the first 24 hours. Each subject had a predose blood sample drawn (time = 0) and took an observed dose of the test or reference drug. Subsequent blood samples were drawn at 2, 4, 6, 8, 10, 12, 24, 48, 72 and 168 hours after the dose [19]. After a 42-day washout period, the subjects took an observed dose of the alternative Alu formulation. Study procedures and blood sampling were repeated as described previously. Each subject had 22 blood samples (3 ml each) drawn over the course of the study for determination of lumefantrine plasma concentrations. The total amount of blood sample collected in the whole study period from each volunteer was 66 ml.

The blood samples were collected in heparinized vacutainers. Each vacutainer was appropriately labeled with Brady number, subject’s identification number (ID), sampling time, sampling hour and date. Blood samples were kept in a cool box and transported within 45 minutes to a BRTC laboratory for centrifugation. The samples were centrifuged at 1800 g at 4°C to obtain plasma samples which were transferred into similarly labeled cryovials. The plasma samples were kept in IHI’s BRTC laboratory at −80°C until transfer to MUHAS-Bioanalytical Laboratory in Dar es Salaam. The samples were carried in a cool box packed with liquid nitrogen. At MUHAS plasma sample were kept at -80°C until the day of analysis.

### Bioanalytics

Blood samples were analysed at MUHAS Bioanalytical laboratory. The plasma analysis for lumefantrine determination was done using an HPLC method with UV detection. The method was developed by ACC laboratory in German in collaboration with MUHAS Bio-analytical Laboratory. The details of the method have been published elsewhere [20].

### Chromatographic conditions

The mobile phase was prepared by dissolving 4.76 g of di-potassium hydrogen phosphate tri-hydrate in 350 ml distilled water. The obtained solution was mixed with 650 ml acetonitrile and the mixture was adjusted to a pH of 3.1 with ortho-phosphoric acid. The pre column (LiChrospher 100) RP 18, 5 μm; 5 × 4 mm and the column (LiChrospher 100) RP18, 5 μm; 125 × 4 mm was used. The flow rate was 1.2 ml/min, detection was achieved at 335 nm and the total run time was 20 min.

### Method validation

The method was validated in which inter-day method; linearity, precision and accuracy were assessed by processing one validation batch each day for three different days. Precision and accuracy of the method was determined using quality control samples spiked at three different concentrations. Low, medium and high quality control samples (QCL, QCM and QCH) were used in three runs performed on different days.

### Pharmacokinetic and statistical analysis of lumefantrine

The pharmacokinetic parameters (C_max,_ T_max,_ AUC_0-168,_ and AUC_0-∞_) of reference and test drugs were compared using ANOVA with period and sequence as within and between subject factors respectively. Analysis was performed in line with FDA bioequivalence guidelines. Bioavailability/bioequivalence was determined using area under the plasma concentration–time curve predose to the last measurable plasma concentration (AUC_0-168_), AUC extrapolated to infinity (AUC_0-∞_), peak plasma concentrations (C_max_) and time required to attain peak concentration (T_max_).

Non-compartmental PK analysis was employed to determine PK profile of lumefantrine of each tablet formulation using R Statistical software version 2:13. The C_max_ and T_max_ were determined directly from plasma concentrations –time curve. AUC_0-168_ was calculated by a combination of linear and logarithmic trapezoidal methods. AUC_0-∞_ was calculated by the summation of AUC_0-168_ and residual AUC (AUC_0-168_ + C_168_/λz), where λz is terminal elimination rate constant. The λz was estimated by performing log-linear regression on the concentration versus time data points that were determined to describe the terminal, linear elimination phase.

Individual pharmacokinetic parameters were natural log-transformed according to FDA recommendations [17,18], and geometric means and standard deviations calculated. The ratio of the test to reference formulation for geometric mean C_max_, AUC_0-t_, and AUC_0-∞_ and the 90% confidence intervals around each mean ratio were determined. Samples below limit of quantitation (LLOQ) were assigned a 50 ng/ml value during estimation of PK parameters. Average bioequivalence would be met if the 90% confidence intervals around the C_max_, AUC_0-168_, and AUC_0-∞_ mean ratios for each drug lied within the FDA's predefined limits of 0.80 to 1.25 [17,18].

## Results

### Volunteers and baseline characteristics

Table 1, describes the demographic characteristics, mean baseline haematology, and biochemistry of the 18 study volunteers who completed the study. Both test and reference drug formulations were well tolerated. There were no adverse drug reactions, abnormal laboratory results or dropouts.

**Table 1:** Volunteers demographic and baseline characteristics (n = 18)

**Parameter**	**Value**
Age (years ± SD)	27 ± 7.3
Weight (Kg ± SD)	67.3 ± 12.6
BMI (Kg/M^2^ ± SD)	22.7 ± 3.0
WBC (X10^3^/μL)	5.98
RBC (X10^6^/μL)	5.082
HGB (g/Dl)	14.61
Albumin (g/L)	41.61

### Lumefantrine method validation

The inter-day accuracy and precision of the method were within the acceptable limits. The coefficients of variation (cv%) for QCL, QCM and QCH samples in all three runs performed on different days fulfilled the acceptance criteria as were always ≤15%. The relative standard deviations from the nominal value for QCL, QCM and CQH in all runs were always ≤15%. A total of 394 test plasma samples were analysed in eight batches and the peaks were well separated

### Bioavailability and mean bioequivalence data

The results of PK parameters are presented as mean ± SD with reference to lumefantrine (Table 2). The primary PK parameters C_max_, T_max_, AUC_0-168_ and AUC_0-∞_ for Artefan® and Coartem® formulations were determined under fed condition. The C_max_ of lumefantrine for both drugs was achieved within 6.22 hours (Table 2). The mean residual AUCs extrapolated to infinity was similar for both formulations with 10.44% for Artefan® and 9.78% for Coartem (P-value = 0.95).

**Table 2:** **% Ratio of untransformed data (Artefan® to Coartem *® *) and a comparison of data obtained by Norvatis**

***Primary Parameter***	***Artefan***^***®***^	***Coartem***^***®***^	***P value***	***% Ratio of test to reference***	***Norvatis Data on file (Riamet)***
	***Arithmetic mean ± SD***	***Arithmetic mean ± SD***			***Arithmetic mean***
	(*)	(*)			
Tmax (h)	6.11±2.698	6.2±2.2	0.8950	-	6 – 8
Cmax (ng/ml)	4206.9±2942.5 (3144.0)	4438.81±2548.432 (3742.267)	0.7836	84.0	5100-9800
AUC_0-168_ (ng.h/ml)	123758.9±83527.5 (93175.97)	135430.7±86814.8 (109729.0)	0.6612	84.5	-
AUC_0-∞_ (ng.h/ml)	138189.7±94959.8 (102441.5)	149530.2±95109.4 (121576.3)	0.7022	84.3	108000- 243000

*Geometric mean.

**Figure 1:**
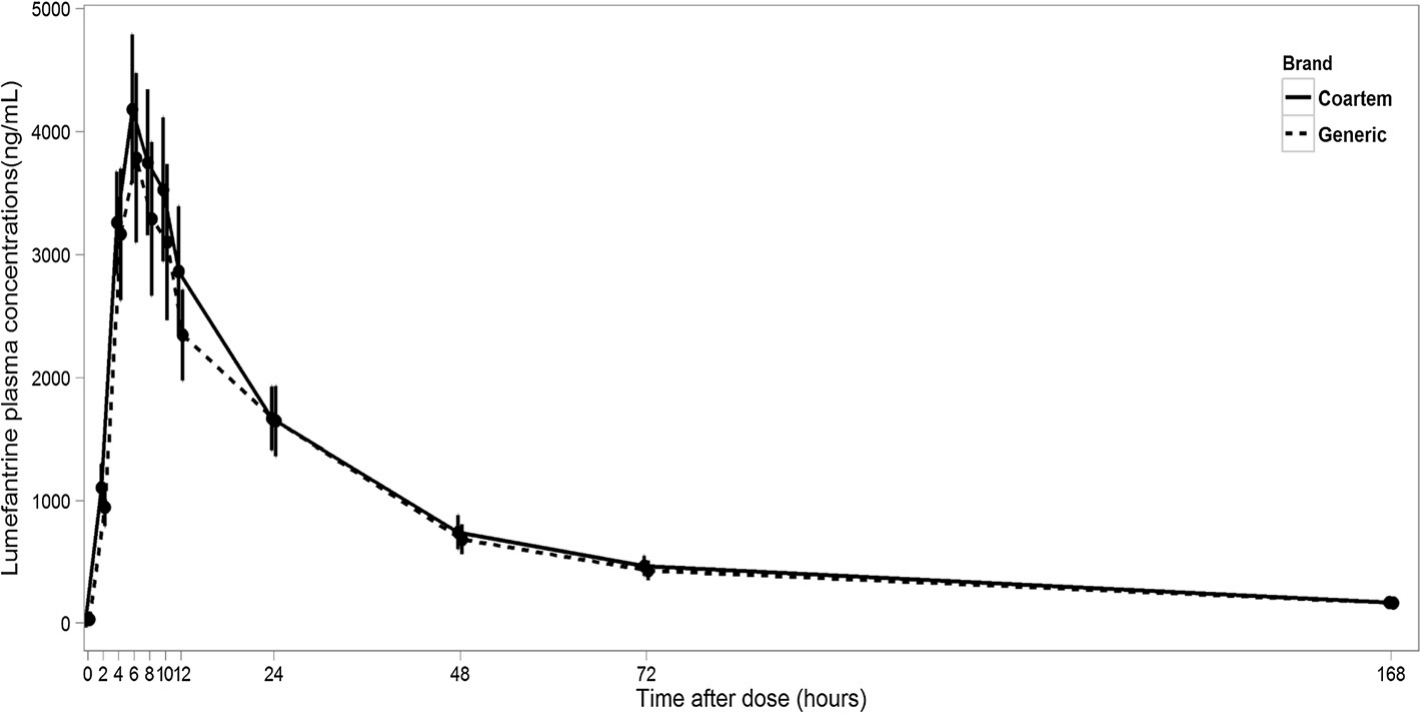
**Mean plasma concentration-time profiles of lumefantrine in 18 health volunteers after oral administration of either a 20/120 mg Coartem® or Artefan® (generic) tablet formulation.** Error- Bars represent mean ± standard error.

Plasma concentrations of lumefantrine attained by administration of a single dose of Artefan® and Coartem® formulations were comparable based on lumefantrine concentration-time profiles for Artefan® and Coartem® formulations (Figure 1).

Mean ratios of Artefan® to Coartem® natural log-transformed C_max_, AUC_0-168_, and AUC_0-∞_ values and 90% confidence interval limits are summarized in Table 3. One out of the 18 subjects had predose lumefantrine concentration above the lower limit of quantitation (50 ng/mL) in period 1 even after the washout period. Other six volunteers had lumefantrine predose concentrations ranging from 50.6 to 221.6 ng/mL. Of the six subjects, four had lumefantrine in period one (55–190 ng/mL) and the rest had lumefantrine (50.6 to 221.6 ng/mL) after the wash out period, and all were included in the lumefantrine pharmacokinetic analysis as concentrations at time zero. However, none of the subjects had a predose concentration greater than 5% of his lumefantrine C_max_ and thus all concentrations were acceptable for inclusion in average bioequivalence analysis in accordance with FDA recommendations.

**Table 3:** Confidence interval from log transformed data for Bioequivalence assessment

***Pharmacokinetic parameters***	***Ratio of Artefan® to Coartem® (%)***	***90% Confidence interval of the geometric mean ratio***	***Acceptance criteria***	***P-value (normal)***
LnC_max_	84.0	49 - 143%	80 - 125%	0.559
LnAUC_0-168_	84.5	53 - 137%	80 - 125%	0.538
LnAUC_0-∞_	84.3	52 - 135%	80 - 125%	0.575

**Figure 2:**
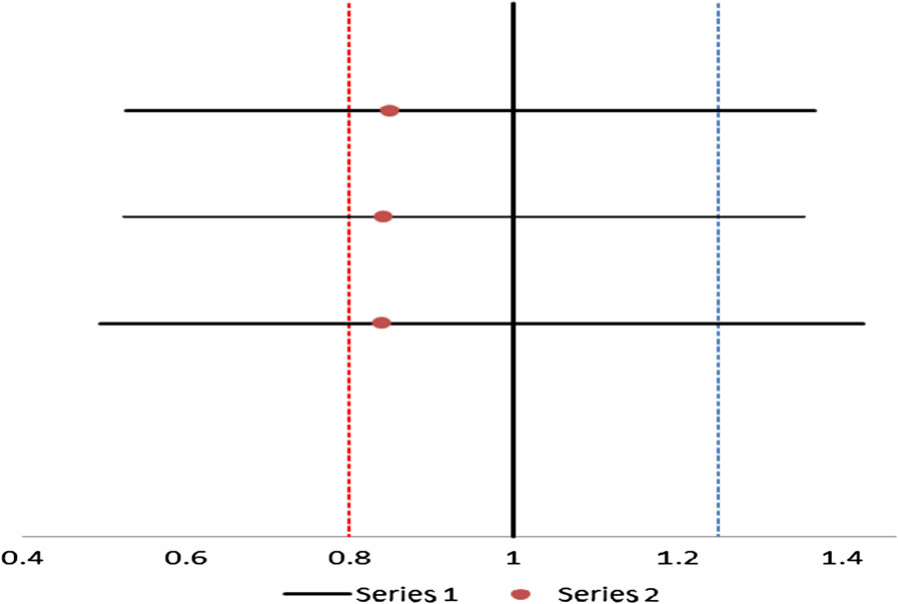
The 90% confidence interval for average bioequivalence measures (Cmax and AUC) plotted over the FDA bioequivalence criteria boundary (The red dotted lines represent the FDA recommended lower and upper limits of the 90% confidence interval for Test/Reference ratio).

Artefan® lumefantrine C_max_, AUC_0-t_ and AUC_0-∞_ geometric means were all less by ~ 16% relative to Coartem®. The required mean geometric ratios of C_max_, AUC_0-t_ and AUC_0-∞_ for fulfilling the FDA criteria for bioequivalence are 0.8 – 1.25 and were met. However, as illustrated by Figure 2, the 90% confidence intervals for lumefantrine C_max_, AUC_0-t_, and AUC_0-∞_ mean ratios were not within 80 -125% and, therefore, FDA criteria for average bioequivalence was not met.

The mean day 7 lumefantrine plasma concentrations obtained from Artefan® and Coartem® were 163 and 171 ng/ml whereas the median values were 102 and 147 respectively 171 ng/ml indicating that the test and reference tablet formulations were comparable.

## Discussion

The obtained mean residual AUC values (≤10%) for both Artefan® and Coartem® with estimated AUC_0-168_ of 80% indicate that the selected sampling scheme was sufficiently long enough to ensure adequate description of the elimination phase of lumefantrine.

The obtained mean PK parameters for Artefan® and Coartem were found to have no statistically significant difference (p> > 0.05). The mean AUC values of lumefantrine for both Artefan® and Coartem® were within range of 108,000 and 243,000 ng.h/ml obtained by Novartis (see Table 2). This means that both Artefan® and Coartem® had comparable bioavailability and would equally achieve comparable therapeutic plasma lumefantrine concentrations.

Lumefantrine day 7 plasma concentration is predictive of the treatment outcome in malaria patients who complete the six doses taken for three consecutive days. Patients with lumefantrine levels below 175 ng/ml on day 7 are more likely to experience recrudescence by day 21 or 28 allowing prediction of treatment failure [21]. In this study, the patients took a single Alu dose (4 tablets) and the mean day 7 lumefantrine levels were 163.00 ±148.01 and 171.82 ±116.59 for Artefan® and Coartem® respectively (p-value = 0.98). Despite taking a single dose versus six doses recommended for non-severe malaria treatment, the mean day 7 plasma concentration values were very close to the cut off point and had no statistical significant difference from the latter (175 ng/ml) indicating that both Artefan® and Coartem® are likely to produce adequate drug plasma concentrations to exert antiplasmodial action. The mean and median day 7 lumefantrine plasma concentrations obtained from Artefan® and Coartem®, respectively, were comparable. However, due to observed high inter-individual variability in lumefantrine plasma concentrations, there is possibility of occurrence of treatment failure or drug toxicity in some individuals treated with either Coartem® or Artefan®.

In assessing bioequivalence between Artefan® and Coartem® it was observed that the geometric mean ratios for C_max_, AUC_0-t_ and AUC_0-∞_ were within the acceptable FDA criteria for bioequivalence of 0.8 – 1.25 (Table 3). The overall lumefantrine pharmacokinetic profiles (pharmacokinetic parameters) for both Artefan® and Coartem® in the healthy Tanzanian volunteers by visual inspection appear to be comparable (see Figure 1).

However, the 90% confidence intervals for lumefantrine (18 subjects) C_max_, AUC_0-168_, and AUC_0-∞_ mean ratios were not within 0.80 to 1.25 (Table 3). Figure 2 is a Forest plot indicating 90% confidence interval limits for the ratios of Cmax, AUC 0–168 and AUC 0-∞ obtained by the study and indicates the required limits in line with FDA requirements for BE studies. As shown in the plot, the two tablet formulations could not meet the FDA criterion for bioequivalence. Failure to comply with the FDA 90% confidence intervals may be due to high variability of lumefantrine plasma concentrations observed among individual subjects.

High inter-individual variability for lumefantrine plasma concentration observed in this study could also be due to difference in the capacity of individuals to handle the drug with respect to cytochrome 3A4 (CYP3A4) as well as inconsistency in the extent of drug presentation in the systemic circulation through the gastro-intestinal tract. CYP3A4/5 is expressed in the human small intestine and liver and contributes to the first pass effect of lumefantrine [22]. Lumefantrine is a substrate of CYP3A4/5 which has been shown to be polymorphic. Therefore, drugs undergoing first pass effect by this isoform are expected to have highly variable pharmacokinetics.

## Conclusion

Although the ratios of AUCs and C_max_ were within the acceptable FDA range, bioequivalence between Artefan® and Coartem® tablet formulations could not be demonstrated due to failure to comply with the FDA 90% confidence interval criteria. It is the failure to comply with 90% confidence interval stipulated by FDA guidelines, which made the two tablet formulations to be bioinequivalent. However, based on the observed total drug exposure (AUCs and day 7 plasma lumefantrine concentrations), Artefan® is likely to produce a similar therapeutic response as Coartem®.

## Competing interests

We declare no competing interest of any kind in this work.

## Authors’ contribution

OMSM, SP and NE conceived the study, and participated in its design, coordination, data analysis and writing of the manuscript. AIM participated in the design, data collection, analysis and gave a support in the writing of the manuscript. SS, JO, MC, SA and MR participated in data collection and contributed in the preparation and writting of the manuscript. All authors read and approved the final manuscript.
